# Characterization of the *Arabidopsis thaliana* E3 Ubiquitin-Ligase AtSINAL7 and Identification of the Ubiquitination Sites

**DOI:** 10.1371/journal.pone.0073104

**Published:** 2013-08-28

**Authors:** Diego A. Peralta, Alejandro Araya, Cristina F. Nardi, Maria V. Busi, Diego F. Gomez-Casati

**Affiliations:** 1 Centro de Estudios Fotosintéticos y Bioquímicos (CEFOBI-CONICET), Universidad Nacional de Rosario, Rosario, Argentina; 2 Instituto de Investigaciones Biotecnológicas, Instituto Tecnológico de Chascomús (IIB-INTECH-CONICET), Universidad Nacional de San Martin, Chascomús, Argentina; 3 Centre National de la Recherche Scientifique and UMR 1332 – Biologie du Fruit et Pathologie, Institute National de la Recherche Agronomique (INRA) Bordeaux Aquitaine, Villenave D’Ornon, France; Instituto de Biología Molecular y Celular de Plantas, Spain

## Abstract

Protein ubiquitination leading to degradation by the proteasome is an important mechanism in regulating key cellular functions. Protein ubiquitination is carried out by a three step process involving ubiquitin (Ub) activation by a E1 enzyme, the transfer of Ub to a protein E2, finally an ubiquitin ligase E3 catalyzes the transfer of the Ub peptide to an acceptor protein. The E3 component is responsible for the specific recognition of the target, making the unveiling of E3 components essential to understand the mechanisms regulating fundamental cell processes through the protein degradation pathways. The *Arabidopsis thaliana* seven in absentia-like 7 (*AtSINAL7*) gene encodes for a protein with characteristics from a C3HC4-type E3 ubiquitin ligase. We demonstrate here that AtSINAL7 protein is indeed an E3 protein ligase based on the self-ubiquitination in vitro assay. This activity is dependent of the presence of a Lys residue in position 124. We also found that higher *AtSINAL7* transcript levels are present in tissues undergoing active cell division during floral development. An interesting observation is the circadian expression pattern of *AtSINAL7* mRNA in floral buds. Furthermore, UV–B irradiation induces the expression of this transcript indicating that AtSINAL7 may be involved in a wide range of different cell processes.

## Introduction

Protein turnover through the ubiquitin-mediated proteasome system plays a pivotal role in many regulatory pathways such as growth, cell differentiation, cell cycle control, stress response and apoptosis [1–3]. First described in *Drosophila melanogaster*, Seven in absentia (SINA) proteins are E3 ubiquitin ligases with a characteristic N-terminal RING (Really Interesting New Gene) finger domain, linked to a conserved C-terminal domain required for oligomerization and binding to target proteins [4]. The *D. melanogaster* SINA regulates photoreceptor differentiation by targeting the transcription factor Tramtrack for proteasomal degradation [5–7]. The RING finger motif is defined as a 40-60 cysteine rich domain coordinating two Zinc ions which can fold into a compact domain comprising a small central β-sheet and an α-helix [8] (http://www.ncbi.nlm.nih.gov/Structure/cdd/cddsrv.cgi?uid=pfam00097).

The RING finger domain from many E3 ubiquitin-ligases is required for interaction with an E2 ubiquitin-conjugation protein, leading to the transfer of ubiquitin to the target protein. E3 ubiquitin-ligases are able to recognize a large number of targets through adaptor proteins, which provides precise functional specificity [9,10]. Besides the proteolytic pathways, protein ubiquitination can also regulate protein functions [11,12], making these enzymes key mediators of post-translational protein regulation. Homologous to the Sina superfamily, which is composed of about 35 highly conserved proteins, have also been found throughout eukaryotes [13,14].

The Arabidopsis genome contains more than one thousand genes encoding for E3 ubiquitin ligases, 469 of them predict proteins presenting one or more of the various types of RING domains [15]. Eighteen Siah homologous, SINA, SINAT1–T5, SINAL1–11 and PEX14 are encoded by the *A. thaliana* genome (www.arabidopsis.org). The participation of Sina in plant resistance to pathogens and plant growth has been described [16,17]. Other studies revealed the existence of a link between hormone response on floral development and the ubiquitination pathway [18]. However, very little is known about the function of Siah protein counterparts in plants.

Several lines of evidence reveal that floral development requires proper mitochondrial function [19–21]. We recently demonstrated that plants with a mitochondrial dysfunction were affected in *Arabidopsis thaliana SINA like 7* (*AtSINAL7*, At5g37890) mRNA levels [22,23]. *AtSINAL7*, predicted to be expressed in 21 plant structures most of the growth stages, seem to be involved in multicellular organismal development such as protein ubiquitination and ubiquitin-dependent protein catabolic process (http://www.arabidopsis.org/servlets/TairObject?id=131882&type=locus). However, the precise function of this protein remains unknown.

We decided to characterize biochemically the *AtSINAL7* encoded protein and to study their expression. AtSINAL7 is a protein characterized by the presence of a canonical C3HC4 RING-type cysteine-rich domain able to coordinates two zinc atoms, homologous to the RING-finger E3 ubiquitin ligase protein, SINA [9]. . Here, we show that AtSINAL7 functions, indeed, as an ubiquitin ligase using a self-ubiquitination assay. We present evidence that this activity depends of a Lys residue. Moreover, we study the expression of *AtSINAL7* in different tissues and during daily light cycle and under UV–B exposure. These results strongly argue that AtSINAL7 is an E3 ubiquitin ligase and suggests that it plays an important role in cell processes, particularly during flower development.

## Results

### Expression and purification of recombinant AtSINAL7

**Figure 1:**
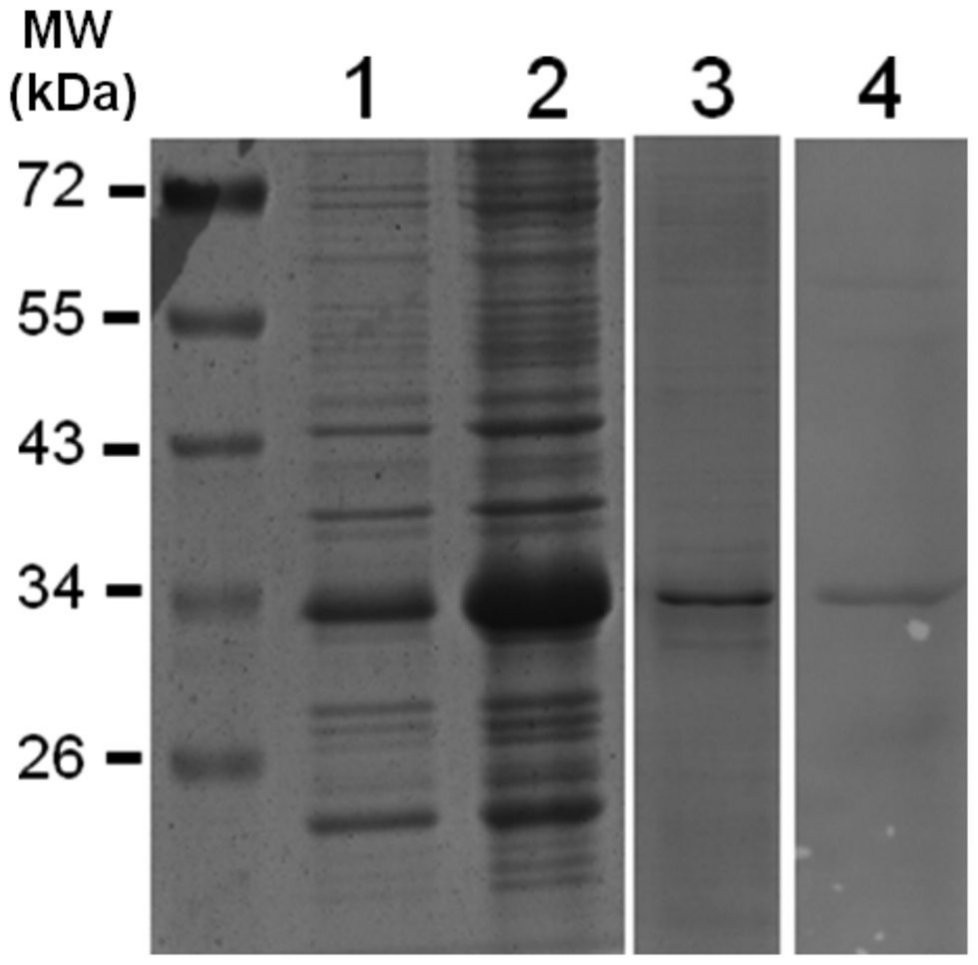
Expression analysis of recombinant AtSINAL7. The *E. coli* cell extracts electrophoresed on SDS-PAGE were revealed with Coomassie brilliant blue staining. Lane 1: Protein extract from uninduced bacteria. Lane 2: Soluble proteins obtained after 5 h IPTG induced bacterial culture. Lane 3: Purified AtSINAL7 fraction stained with Coomassie blue. Lane 4: Western blott analysis of the purified AtSINAL7 fraction revealed using anti-His antobodies. MW (kDa), PageRuler Prestained Protein Ladder (Fermentas).

To characterize the seven in absentia *like* 7 protein from *Arabidopsis thaliana*, the DNA fragment containing the AtSINAL7 coding sequence (286 codons) was fused to an N-terminal His6-tag when cloned onto pRSETb expression vector. The recombinant protein was purified using a HisTrap chelating affinity chromatography after expression in *E. coli* (BL21) pLys strain. The purified recombinant AtSINAL7 protein of 32 KDa was successfully induced (Figure 1, lane 2) and purified to homogeneity as shown by protein staining (lane 3). The presence of recombinant AtSINAL7 was assessed by Western blot analysis using with the anti-His antibody (lane 4).

### Transcript levels of *AtSINAL7* in different tissues of Arabidopsis

**Figure 2:**
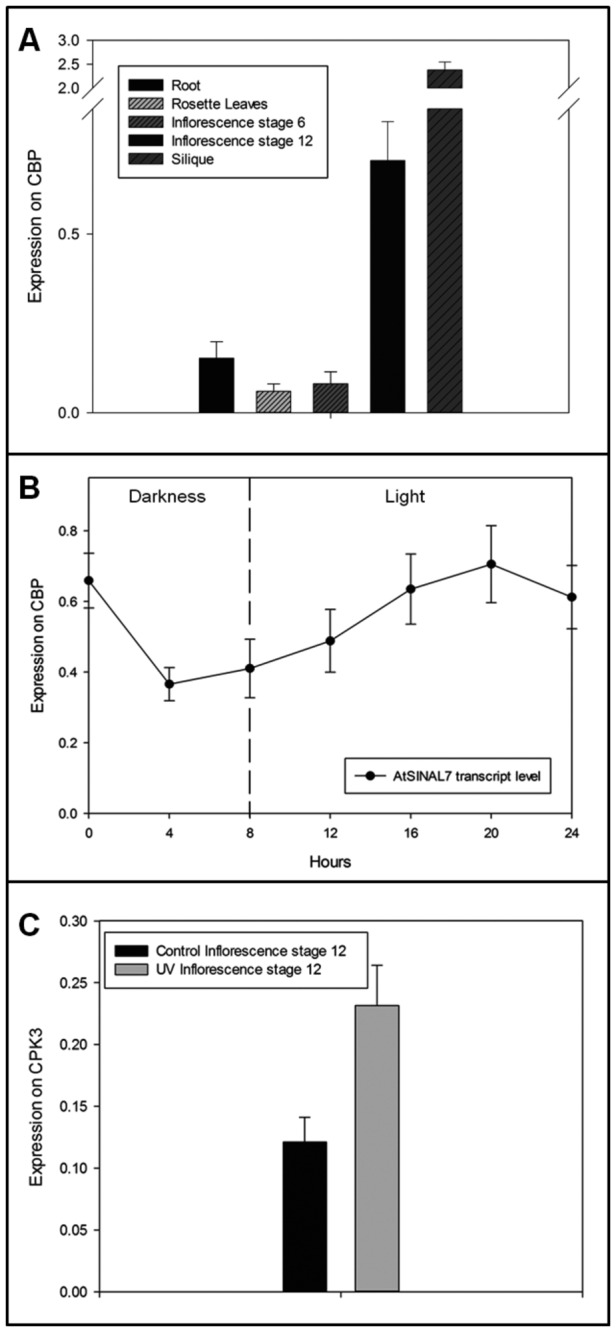
Analysis of AtSINAL7 mRNA levels (A) AtSINAL7 transcript expression profile in different organs from *A. thaliana*. AtSINAL7 transcript levels relative to CBP control gene expression were determined by RT-qPCR in several *A. thaliana* organs. Data shown represent at least three independent experiments (significant statistical difference was determined using t test, P <0.05). **(B) Time-course expression of AtSINAL7 transcripts.** Determination of the transcription levels of *AtSINAL7* at 0, 4, 8, 12, 16, 20, and 24 hours under long day growth conditions. Transcripts levels are plotted as relative values using the cap binding protein (At5g44200) mRNA as an internal control. Data shown represent at least three independent experiments (significant statistical difference was determined using t test, P <0.05). **(C) Effect of the UV–B treatment on the AtSINAL7 transcript levels in wild type *A. thaliana* inflorescences.** The *AtSINAL7* transcript expression in *A. thaliana* inflorescences (stage 12) relative to *cpk3* (calcium-dependent protein kinase 3, At4g23650) was determined after 4 h of UV–B exposure. Control plants were protected from UV–B irradiation using polyester filters (see Methods). Data shown represent at least three independent experiments (significant statistical differences determined using t test, P <0.05).

The expression of *AtSINAL7* was determined in several tissues from wild-type *Arabidopsis* by qRT-PCR (Figure 2A). RNA from Root, Rosette leaves, Inflorescence stage 6, Inflorescence stage 12, and Silique were isolated from 28 days old *A. thaliana* Col *0* plants grown under long-day condition greenhouse (see Methods). cDNA was synthesized from total RNA and was quantified spectrophotometrically. Identical amounts of different cDNA samples were used for qPCR amplifications using *Arabidopsis* sina *like 7* with specific primers (Table 1). The data presented concern relative values obtained from the average of three biological and technical replicates using the *cbp* gene (cap binding protein At5g44200) as a house-keeping control [24] (Figure 2A). *AtSINAL7* transcript levels are highly expressed in siliques (2.3770) and stage 12 inflorescences (0.7053), and roots (0.1524), being lower at Stage 6 inflorescences (0.0807) and rosette leaves (0.0600). Thus, *AtSINAL7* seems to be expressed in a tissue-specific manner with a strong induction of 12 and 40 times in stage 12 flowers and silique respectively, compared to rosette leaves.

**Table 1:** Primers Used in qPCR experiments.

Gene	Primer Name	Secuence
At5g44200	*CBPFw*	CCG GCC TAT TCG TGT GGA TTT TGA
At5g44200	*CBPRv*	CAT AAT TCG TTG GCG CAG CTT GAG
At4g23650	*CPK3Fw*	AAT CCA CGG ATG ATT TAG CA
At4g23650	*CPK3Rv*	ATC TGG AGT GCT GGT GTG AT
At5g37890	*RTAtSINAL7Fw*	GCT ACG AAG CTT TCA CAA TTC C
At5g37890	*RTAtSINAL7Rv*	GTA CAG ATC CTT GTA TGA GCT A

### *AtSINAL7* gene expression show a light-dependent behavior in stage 12 flowers

Flowering behavior is strongly dependent from different environmental signals, particularly light. Day length, a major regulator of flowering, allows sexual reproduction to proceed at an appropriate time [25]. As the higher expression was obtained in floral tissues, a time course profile of the study of *AtSINAL7* transcript levels was performed on *Arabidopsis* stage 12 inflorescences throughout 24 hours under long day growth conditions (see Methods). The expression of *cbp* gene (At5g44200) was used as an internal standard ( [24]). The mRNA levels decrease after 4 h darkness reaching a minimum value (0.365). At the beginning of daylight, the mRNA levels increase to reach a maximum value (0.7053) at 20 hours of the experimental period (12 h after light onset). Thus, a two-fold variation of AtSINAL7 transcript levels is observed over a daily basis (Figure 2B).

### *AtSINAL7* transcript levels increase after UV–B treatment

Ubiquitination plays an important role in DNA damage signal amplification. Upon UV-induced DNA damage, several ubiquitin-tagged proteins are degraded by proteasome [26]. To investigate the behavior of AtSINAL7 gene in plants under UV–B exposure, we determined the transcript levels by qRT-PCR at stage 12 inflorescence. The experiment was carried out as follows: wild type Arabidopsis plants were grown in a long-day greenhouse during 4 weeks and then irradiated with UV–B light at 16 hours (8h dark plus 8h light) after the onset of the experiment. Control plants, present in the same environment, were protected with a plastic membrane able to exclude the UV–B light. The inflorescence stage 12 cDNA from control and UV-treated plants were submitted to qRT-PCR analysis (Figure 2C) using cpk3 (calcium-dependent protein kinase 3, At4g23650) as a house-keeping control gene whose expression remains unchanged after UV–B treatment [27]. The results allowed us to conclude that SINAL7 gene expression augments almost 2-fold in inflorescences after UV–B treated plants. Similar results were obtained in rosette leaves, (data not shown), proving that UV–B irradiation triggers *AtSINAL7* transcript expression.

### AtSINAL7 undergoes self-ubiquitination

**Figure 3:**
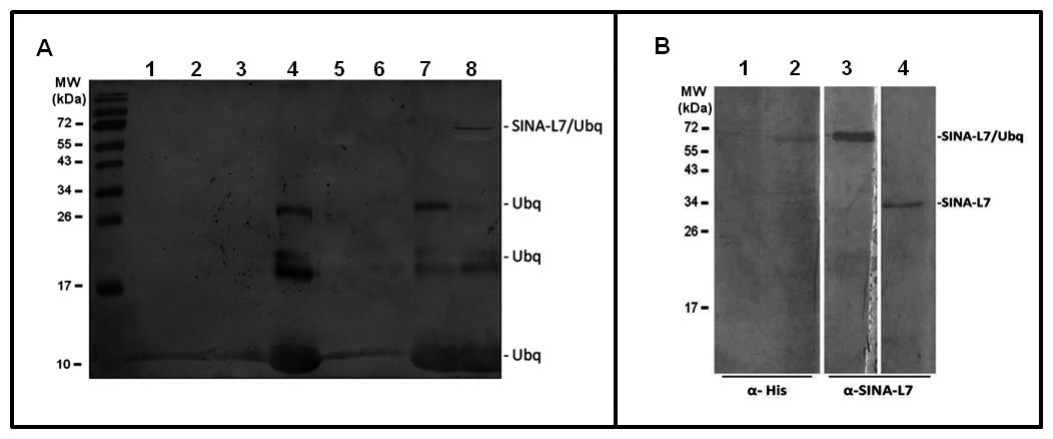
AtSINAL7 E3 ligase activity and identification of self-ubiquitinated AtSINAL7. **(A) E3 ligase activity of AtSINAL7.** *In vitro* self-ubiquitination reactions were performed using cMyc-ubiquitin as a substrate as described in the Methods section. Lane 1: Ubiquitination buffer. Lane 2: Ubiquitination buffer + E1. Lane 3: Ubiquitination buffer + E2. Lane 4: Ubiquitination buffer + cMyc-ubiquitin. Lane 5: Ubiquitination buffer + AtSINAL7. Lane 6: Ubiquitination buffer + E1 + E2. Lane 7: Ubiquitination buffer + E1 + E2 + cMyc-ubiquitin. Lane 8: Ubiquitination buffer + E1 + E2 + cMyc-ubiquitin + AtSINAL7. AtSINAL7/ubiquitin conjugates were resolved by electrophoresis on 15% (w/v) SDS-PAGE and detected by immunoblot analysis using anti-cMyc antibody. **(B) Identification of self-ubiquitinated AtSINAL7.** AtSINAL7/ubiquitin conjugates were electrophoresed on 15% (w/v) SDS-PAGE and detected by immunoblot analysis using anti-HIS (Qiagen) and polyclonal anti-AtSINAL7. Lane 1: Control containing the ubiquitination assay buffer alone. Lane 2 and 3: purified His-AtSINAL7 incubated in the complete ubiquitination reaction medium containing the cMyc-ubiquitin substrate and the yeast E1 and UbcH2 E2 components. Lane 4: purified His-AtSINAL7 alone. MW (kDa), PageRuler Prestained Protein Ladder (Fermentas).

The *in vitro* ubiquitination assay was carried out using heterologous partners: a yeast ubiquitin-activating (E1) enzyme and the human recombinant UbcH2 ubiquitin-conjugating (E2) enzyme, in the presence of cMyc-tagged ubiquitin (cMyc-ubiquitin) as described in the Methods section. Purified recombinant AtSINAL7 was used as ubiquitin-ligating (E3) enzyme. As expected, no signal was detected in lanes lacking cMyc-ubiquitin (Figure 3A, lanes 1, 2, 3, 5, and 6), indicating that the anti-cMyc antibodies do not cross react with other protein components present in the reaction mixture. Lanes 4 and 7, containing only the tagged ubiquitin and the reaction mixture lacking the E3 component respectively, show four bands characteristic of the monomer (9.3 kDa) and oligomers corresponding to cMyc-ubiquitin. Interestingly, the complete reaction mixture shows a protein band corresponding to ubiquitinated AtSINAL7 migrating with an apparent molecular weight approximately 72 kDa (Figure 3A, lane 8). To assess that AtSINAL7 was indeed self-ubiquitinated the samples 5 and 8 containing the E3 component alone and the complete reaction mixture respectively were analyzed by Western blot using anti-His6 (Santa Cruz Biotechnology) and anti-recombinant AtSINAL7 antibodies (Figure 3B). The same band is revealed by both anti-His and anti-AtSINAL7 (Figure 3B, lanes 2 and 3). It is interesting to note that AtSINAL7 identified by anti-AtSINAL7 antibody (lane 3) migrates at 34 kDa (lane 4) while the self-ubiquitinated form show a mobility shift to near 70 kDa, suggesting that more than one ubiquitin molecules is has been incorporated to the AtSINAL7 protein.

### The residue K124 is involved in self-ubiquitination of AtSINAL7

**Figure 4:**
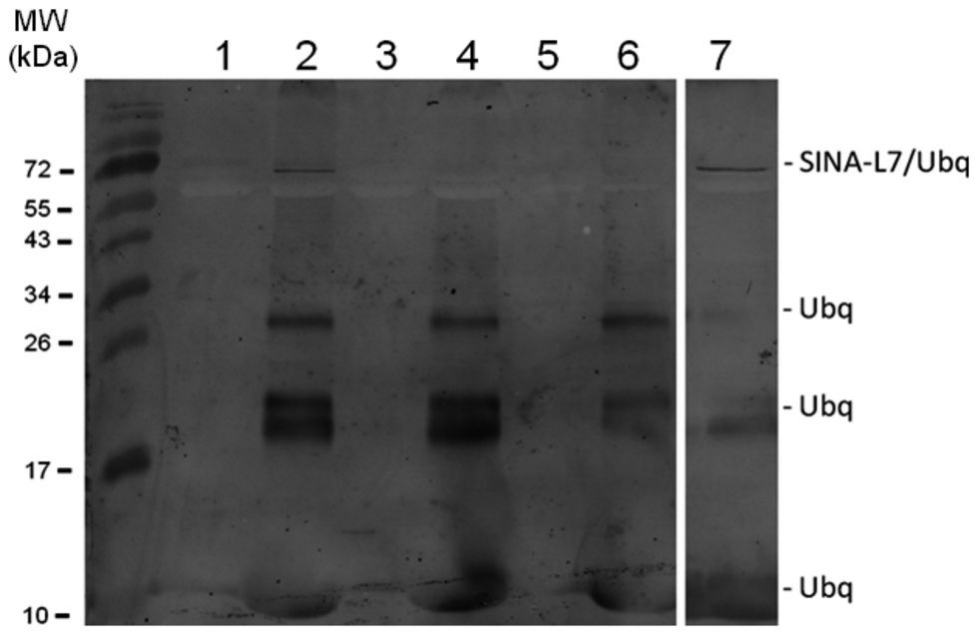
E3 ligase activity of AtSINAL7 mutants. *In vitro* self-ubiquitination reactions were performed incubating purified His-SL7K23A (lane 2), His-SL7K124A (lane 4), His-SL7K23AK124A (lane 6) and His-AtSINAL7 (lane 7) in the presence of cMyc-ubiquitin, yeast E1, E2 UbcH2 as described in the Methods section. Reaction products were separated on 15% (w/v) SDS-PAGE and ubiquitinated proteins were detected by immunoblot analysis using anti-cMyc antibody. MW (kDa), PageRuler Prestained Protein Ladder (Fermentas).

To determine the residues involved in the AtSINAL7 self-ubiquitination, *in silico* analysis was conducted using the program UbPred: Predictor of protein ubiquitination sites [28]. Two out of 19 Lys, residues on AtSINAL7 were predicted as susceptible to accept the ubiquitin molecule by the bio-computer analysis (http://www.ubpred.org/), at position K23 and K124. To confirm this hypothesis, we construct mutant proteins where the residues K23 and K124 were replaced by alanine (see methods). Three constructs K23A, K124A and the double mutant K23A/K124A were used to produce the recombinant proteins SL7K23A, SL7K124A and SL7K23AK124A for *in vitro* ubiquitination experiments (Figure 4). Only the mutant SL7K23A was able to sustain self-ubiquitination (Figure 4, lane 2) which is comparable to result obtained with the wild-type control (Figure 4, lane 7). In contrast, the mutant SL7K124A (lane 4) and the double mutant SL7K23AK124A (Figure 4, lane 6) were unable to underwent self-ubiquitination.

### Mutations K23A and K124A do not affect protein folding

**Figure 5:**
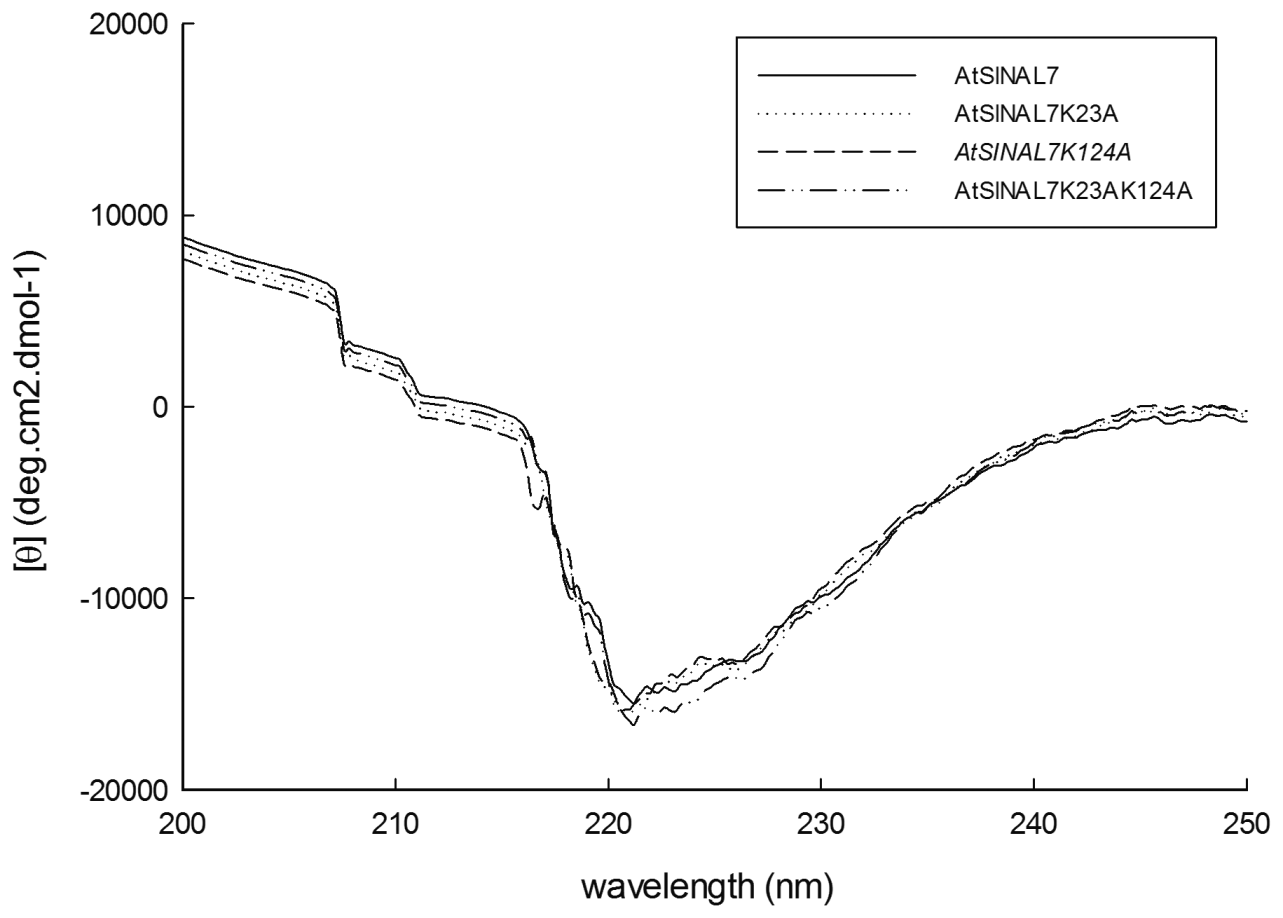
Far-UV CD Spectra of recombinant AtSINAL7 and AtSINAL7 mutants. Far-UV CD spectra were obtained using a Jasco J-810 spectropolarimeter (Jasco International Co.) over the wavelength range from 190 to 250 nm, at 25°C. Measurements were performed in a 0.2 cm quartz cuvette at a rate of 100 nm.min^-1^, bandwidth of 1 nm, response time of 2 s, data pitch of 1 nm, and accumulation of 10. CD data are shown as the mean residue ellipticity (deg.cm2.dmol^-1^) obtained after subtracting the baseline, smoothing, and data normalization. CD spectra for AtSINAL7 (solid line), AtSINAL7K23A (dotted line), AtSINAL7K214A (dashed line) and AtSINAL7K23AK124A (dashed and dotted line) were recorded in 20 mM Na-phosphate buffer, pH 7.4.

Since the mutation of Lys residues may induce structural changes on the protein, we decided to study the secondary structure of the recombinant AtSINAL7 and the respective K-to-A mutants. The correct folding of recombinant AtSINAL7(K23A), AtSINAL7(K124A) and AtSINAL7(K23AK124A) proteins was analyzed using circular dichroism (CD) (Figure 5). The percentage of the secondary structure was estimated from CD spectra using the K2D algorithm [29]. The content of alpha helix, beta sheet and random coil were 26.76, 30.03 and 43.21% respectively for the recombinant AtSINAL7. Interestingly, all three mutants presented identical CD spectra to the wild type AtSINAL7 protein, indicating that no changes on the secondary structure were induced when the residues K23, K124 or both simultaneously were changed for Ala on the protein. Considering that the average error observed from CD spectra was lower than the values obtained in secondary structure prediction using K2D (0.08; 0.09), we conclude that the lack of self-ubiquitination of AtSINAL7(K124A) and AtSINAL7(K23AK124A) was not resulting from a loss of secondary structure, but to the replacement of the Lys-124 residue.

## Discussion

AtSINAL7 is a RING-finger protein homologous to the Drosophila´s *seven* *in absentia* (sina) protein. The Drosophila sina superfamily possesses important functions in development control and protein degradation by the proteasome. These proteins are an essential part of the ubiquitin protein modification machinery found in all eukaryotic organisms [9,18]. This particular protein modification participates in many different cell process by affecting gene expression through the protein turnover pathways or by modification of the protein function [2]. The important role played by these proteins is resulting from both the E3 ubiquitin ligase activity, and the capacity of E3 ligases to recognize specifically the target protein. Thus, the knowledge of E3 RING-finger ubiquitin ligases is crucial to understand the physiological mechanisms involved in plant development and adaptation.

Eighteen genes homologous to Siah from Sina superfamily, SINA, SINAT1–T5, SINA *like* 1–11, and PEX14 are present in the *Arabidopsis* genome (www.arabidopsis.org). Several functions have been reported for plant Sina genes such as resistance to pathogens, plant growth [16,17], hormone response and floral development [18]. Interesting, floral development depends on proper mitochondrial function. Consistent with this observation, we found that mitochondrial dysfunction affected *AtSINAL7* expression in young flowers [22,23].

Here, we show that *AtSINAL7* transcripts are more abundant in tissues engaged in active cell division in different organs of *A. thaliana*, with higher levels in flowers and siliques (Figure 2A), these results are in agreement with the transcript level pattern shown in *Arabidopsis* eFP Browser at bar.utoronto.ca [30]. An important result of this study is the fact that the transcript levels oscillate daily with the lower level at 4 hours in dark, and attains the higher levels after 12 hours of light onset in a long-day growing cycle. This behavior is in agreement with the presence of putative circadian control boxes in the promoter region of the *AtSINAL7* gene, predicted by using the Genomatix Software Suite (http://www.genomatix.de/solutions/genomatix-software-suite.html). In addition, an increase of *AtSINAL7* level was induced by UV–B irradiation in *Arabidopsis* . During DNA damage response generated by UV light, several proteins undergo polyubiquitylation to be processed by the proteasome [26], indicating that important transcriptional and metabolical processes are affected by UV–B irradiation [31]. It is interesting to note that the expression of the multifunctional E3 ubiquitin ligase, COP1 gene, is controlled by a combinatorial regulation of FHY3 and HY5 in response to UV–B [32]. Thus, considering that AtSINAL7 has the signature of a RING-type E3 ubiquitin ligase, the increase of transcript upon UV–B exposure is in agreement with a potential role in complex signalling pathways.

We demonstrate that SINAL7 is able to participate as an ubiquitin ligase (E3 component) in an E1-E2-E3 assay (Figure 3A). Moreover, we found that the Lys124, located in the sina domain, is required for this function. However, we tested this function in a self-modification assay; if this residue acts as an acceptor of ubiquitin, plays a role in catalysis or both, remains to be elucidated.

Self-ubiquitination of E3 ubiquitin ligases has been proposed to regulate their activity, the recruitment of substrates and to participate in non-catalytic functions of these proteins [1,33]. Considering that AtSINAL7 contains a TRAF-like domain, self-ubiquitination may be a mechanism to recruit substrates with ubiquitin-ubiquitin properties as shown for the TRAF6, a RING domain E3 ligase that has a crucial role in the initial activation of signaling cascades [34–36]. Thus, it is possible to speculate that AtSINAL7 undergo self-ubiquitination as a target for degradation by the proteasome complex, but also to accomplish different activities in plant cells. *In vivo* studies will be necessary to validate this hypothesis.

## Conclusions

We show here that AtSINAL7, a predicted RING-domain ubiquitin E3 ligase, is able of undergo self-ubiquitination *in vitro*. This activity is dependent of the presence of a Lys residue in position 124. In addition, we show that *AtSINAL7* transcript levels are high in *A. thaliana* tissues undergoing active cell division, suggesting a role during floral development. An interesting observation is the circadian expression pattern followed by the *AtSINAL7* mRNA levels. Furthermore, UV–B irradiation induces the expression of this transcript indicating that this protein may be involved in a wide range of different cell processes.

## Materials and Methods

### Plant Material and Bacterial Strains

*A. thaliana* (var. Columbia Col-0), grown in a greenhouse under long day conditions (16 day/8 night), were used in this study. *Escherichia coli* BL21(DE3) pLysS strain (*E. coli B F–dcm ompT hsdS(rB–mB–) gal λ(DE3) [pLysS Camr]*) was used as bacterial hosts in cloning and expression experiments.

### Cloning and Expression of AtSINAL7

The total RNA extracted from Arabidopsis leaves was used as template for cDNA synthesis using random hexamers. The cDNA fragment (860bp) containing the *AtSINAL7 coding region* was PCR amplified using GoTaq Polymerase (Promega, WI, USA) and the following primers: SINAL7up: 5´-AAAGGATCCAATGGGTGCCGCGATTTTG-´3 and SINAL7down: 5´-CTCGAGTTACTCTTTGTTCAACTTCTTGAC-´3. The PCR product was cloned into pGEMT-Easy (Catalog# A1360 Promega, WI, USA). The AtSINAL7 coding sequence was obtained by double digestion with XhoI and BamHI restriction enzymes from the recombinant plasmid. The purified fragment was inserted into pRSETb expression plasmid (Invitrogen, Carlsbad, CA, USA), previously digested with the same restriction enzymes, using TDNA ligase (Promega, WI, USA). The resulting recombinant plasmid, pRSETbSL7, containing 286 codons from the *Arabidopsis thaliana* seven in absentia *like* 7 (AtSINAL7) containing a N-terminal His-tag sequence, under the control of phage T7 promoter, was used to transform *E. coli* BL21 (DE3) pLysS cells

### Purification of AtSINAL7, SL7K23A, SL7K124A and SL7K23AK124A

*E. coli* BL21 (DE3) pLysS cells harboring plasmid pRSTEbSL7 were grown at 37°C in TB medium, containing 100 mg ml^-1^ ampicillin to an OD_600_ = 0.6. AtSINAL7 production was induced by the addition of 1 mM IPTG and subsequent incubation at 30°C for 8 h. Cells were harvested and re-suspended in 20 mM Tris-HCl, pH 7.4, containing 1 mM phenylmethylsulfonyl fluoride (PMSF), disrupted by sonication and centrifuged at 7000 x g for 15 min at 4°C. The supernatant was loaded onto a HiTrap chelating column (GE Healthcare). After washing with 20 ml of 20 mM Tris-HCl, pH 7.4, 20 mM imidazole, the recombinant protein was eluted using a 20–500 mM imidazole gradient in buffer 20 mM Tris-HCl, pH 7.4. The elution of the recombinant protein was monitored by enzyme activity and SDS–PAGE analysis of chromatography fractions. The purified enzyme was pooled and concentrated to >1 mg ml^-1^ and used immediately after purification process.

### Protein analyses

SDS–PAGE was performed using 12% (w/v) gels as described by Laemmli [37]. Gels were revealed by Coomassie blue staining or after electro-blotting onto nitrocellulose membranes (Bio-Rad). Membranes were incubated with penta-His antibody (Qiagen) or polyclonal anti-AtSINAL7 antibodies. The antigen–antibody complex was visualized with alkaline phosphatase-linked anti-mouse IgG or anti-rabbit IgG, followed by staining with BCIP and NBT [38]. Total protein concentration was determined as described by Bradford [39].

### E3 ubiquitin ligase activity assay

*In vitro* ubiquitination assay was adapted from the protocol described by Wertz et al. [40]. The reaction mixture (25 μl) contained 20 mM HEPES, 100 mM NaCl, 5 mM MgCl2, 5 mM ATP, 10 mM DTT, 5 μg cMyc-ubiquitin (Bostom Biochem), 150 nM yeast E1 (Bostom Biochem), 200 mM human recombinant UbcH2 (Bostom Biochem) and 5 μg of purified His-AtSINAL7. The reaction mixture was incubated at 30°C. After 2 h, the reaction was stopped by adding 5X SDS-PAGE Sample buffer (125 mM Tris-HCl pH 6.8, 20% Glycerol, 4% SDS and 10% β-mercaptoethanol) and boiled at 100°C for 5 min. Protein samples were analyzed by SDS-PAGE electrophoresis followed by protein gel blotting. Blots were probed using anti-cMyc antibodies (Bostom Biochem), followed by incubation with anti-mouse Alkaline Phosphatase conjugated antibodies (Sigma).

### RNA Preparation and Quantitative Real-Time PCR

Total RNA was isolated from 30 mg of tissue using SV Total RNA Isolation System (Promega, WI, USA) as described in the manufacturer’s protocol. cDNA synthesized using 5X M-MLV buffer (250 mM Tris-HCl, 375 mM KCl, 15 mM MgCl2, 50mM DTT), dNTPs MIX (dATP, dCTP, dGTP and dTTP 10 mM each), 2 µg/µL random hexamers pd(N) 6 (Amershan #27-2166-01) as primers, Recombinant RNASin^®^ Ribonuclease Inhibitor (25 units), and 200 units of MMLV reverse transcriptase (USB Corp., Cleveland, OH, USA), incubating 1 h at 37 °C. After quantification by UV absorption at 260 nm, 2 µg of cDNA was used as a template for qPCR amplification in a MiniOPTICON2 apparatus (Bio-Rad), using the intercalation dye SYBRGreen I (Invitrogen) as a fluorescent reporter and GoTaq Polymerase (Promega). Primers, able to amplify unique 150-200 bp products, were designed using the online primer design tool Primer-BLAST (http://www.ncbi.nlm.nih.gov/tools/primer-blast/) (Table 1). Amplification conditions were : 2 min denaturation at 94°C; 40–45 cycles at 94°C for 15 s, 57°C for 20 s, and 72°C for 20 s; followed by 10 min extension at 72°C. Three technical replicates were performed for each sample. RNA from each sample was obtained from pools of at least three plants. Melting profile for each PCR was determined by measuring the decrease of fluorescence with increasing temperature (from 65°C to 98°C). The size of the amplification products was verified on 2% (w/v) agarose gel. Gene expression was normalized to the *Arabidopsis* cbp (cap binding protein, At5g44200) house-keeping gene [24] and *Arabidopsis* cpk3 (calcium-dependent protein kinase 3, At4g23650) house-keeping gene [27] in UV–B treatment experiments.

### Site-directed Lys-to-Ala SINAL7 mutants

The codons for Lys 23 and Lys 124 on the AtSINAL7 coding region were changed to Ala triplets using the QuickChange II XL site-directed mutagenesis kit (Stratagene, La Jolla, CA, USA). The pRSETbSL7 vector was used as template for PCR amplification. The primers used were: SL7K23A, GATCTAACAGCATTCTCTCGCAAgcGAGACAACTTTCTTCTAGTGAT; SL7K124A, TTGCAAAAAGAATGTATCTTATGGGgcAGAGTTAACTCATGAAAAGGAATGC, and their respective complementary oligonucleotides. Base substitutions are indicated by lower-case letter. The resultant, single, SL7K23A, SL7K124A, and double SL7K23AK124A mutant vectors were verified by DNA sequencing and used to transform E. coli BL21 (DE3) pLysS cells.

### UV–B treatment of Arabidopsis plants

UV–B irradiation of plants was performed as described by Lario [41]. Arabidopsis plants were exposed for 4 h to UV–B radiation in a growth chamber using UV–B bulbs (Bio-Rad, Hercules, CA, USA). UV–B lamps were covered with cellulose acetate filters to exclude the wavelengths below 280 nm (100 mm extra clear cellulose acetate plastic; Tap Plastics, Mountain View, CA, USA) and placed 30 cm above the plants. The UV radiation measured with a UV–B/UVA radiometer (UV203 AB radiometer; Macam Photometrics, Scotland, UK) was 2 W m^-2^ for UV–B and 0.65 W m^-2^ for UV-A. Control plants, were exposed for the same period of time to the light sources described above covered with a polyester filter (100 µm clear polyester plastic; Tap Plastics) to absorbs both UV–B (0.04 W m^-2^) and wavelengths <280 (UV-A radiation intensity was 0.4 W m^-2^). Immediately after irradiation, samples from at least three independent biological replicates were collected, frozen in liquid nitrogen, and stored at -80°C until its use for RNA isolation.

### Circular Dichroism (CD) studies

Far-UV CD spectra were obtained using a Jasco J-810 spectropolarimeter (Jasco International Co.) over the wavelenth range from 200 to 250 nm, at 25°C. Measurements were performed in a 0.2 cm quartz cuvette at rate of 100 nm.min^-1^, bandwidth of 1 nm, response time of 2 s, data pitch of 1 nm, and accumulation of 10. CD data are shown as the mean residue ellipticity (deg cm^2^ dmol^-1^) obtained after subtracting the baseline, smoothing, and data normalization. CD spectra for AtSINAL7, AtSINAL7 K23A, AtSINAL7 K124A and AtSINAL7 K23AK124A (0.1-1 mg ml^-1^) were recorded in 20 mM Sodium phosphate buffer, pH 7.4. Secondary structure analysis from CD spectra data was performed using the K2d algorithm [29].

## References

[1] de Bie P, Ciechanover A (2011) Ubiquitination of E3 ligases: self-regulation of the ubiquitin system via proteolytic and non-proteolytic mechanisms. Cell Death Differ 18: 1393-1402. doi:10.1038/cdd.2011.16. PubMed: 21372847. 21372847 10.1038/cdd.2011.16 PMC3178436

[2] Komander D, Rape M (2012) The ubiquitin code. Annu Rev Biochem 81: 203-229. doi:10.1146/annurev-biochem-060310-170328. PubMed: 22524316. 22524316 10.1146/annurev-biochem-060310-170328

[3] Teixeira LK, Reed SI (2013) Ubiquitin Ligases and Cell Cycle Control. Annu Rev Biochem, 82: 387–414. PubMed: 23495935. 23495935 10.1146/annurev-biochem-060410-105307

[4] Hu G, Fearon ER (1999) Siah-1 N-terminal RING domain is required for proteolysis function, and C-terminal sequences regulate oligomerization and binding to target proteins. Mol Cell Biol 19: 724-732. PubMed: 9858595. 9858595 10.1128/mcb.19.1.724 PMC83929

[5] Li S, Li Y, Carthew RW, Lai ZC (1997) Photoreceptor cell differentiation requires regulated proteolysis of the transcriptional repressor Tramtrack. Cell 90: 469-478. doi:10.1016/S0092-8674(00)80507-3. PubMed: 9267027. 9267027 10.1016/s0092-8674(00)80507-3

[6] Carthew RW, Rubin GM (1990) seven in absentia, a gene required for specification of R7 cell fate in the Drosophila eye. Cell 63: 561-577. doi:10.1016/0092-8674(90)90452-K. PubMed: 2146028. 2146028 10.1016/0092-8674(90)90452-k

[7] Cooper SE (2007) In vivo function of a novel Siah protein in Drosophila. Mech Dev 124: 584-591. doi:10.1016/j.mod.2007.04.007. PubMed: 17561381. 17561381 10.1016/j.mod.2007.04.007

[8] Lorick KL, Jensen JP, Fang S, Ong AM, Hatakeyama S et al. (1999) RING fingers mediate ubiquitin-conjugating enzyme (E2)-dependent ubiquitination. Proc Natl Acad Sci U S A 96: 11364-11369. doi:10.1073/pnas.96.20.11364. PubMed: 10500182. 10500182 10.1073/pnas.96.20.11364 PMC18039

[9] Deshaies RJ, Joazeiro CAP (2009) RING domain E3 ubiquitin ligases. Annu Rev Biochem 78: 399-434. doi:10.1146/annurev.biochem.78.101807.093809. PubMed: 19489725. 19489725 10.1146/annurev.biochem.78.101807.093809

[10] Lipkowitz S, Weissman AM (2011) RINGs of good and evil: RING finger ubiquitin ligases at the crossroads of tumour suppression and oncogenesis. Nat Rev Cancer 11: 629-643. doi:10.1038/nrc3120. PubMed: 21863050. 21863050 10.1038/nrc3120 PMC3542975

[11] Sun L, Chen ZJ (2004) The novel functions of ubiquitination in signaling. Curr Opin Cell Biol 16: 119-126. doi:10.1016/j.ceb.2004.02.005. PubMed: 15196553. 15196553 10.1016/j.ceb.2004.02.005

[12] Behrends C, Harper JW (2011) Constructing and decoding unconventional ubiquitin chains. Nat Struct Mol Biol 18: 520-528. doi:10.1038/nsmb.2066. PubMed: 21540891. 21540891 10.1038/nsmb.2066

[13] Della NG, Senior PV, Bowtell DD (1993) Isolation and characterisation of murine homologues of the Drosophila seven in absentia gene (sina). Development 117: 1333-1343. PubMed: 8404535. 8404535 10.1242/dev.117.4.1333

[14] Holloway AJ, Della NG, Fletcher CF, Largespada DA, Copeland NG et al. (1997) Chromosomal mapping of five highly conserved murine homologues of the Drosophila RING finger gene seven-in-absentia. Genomics 41: 160-168. doi:10.1006/geno.1997.4642. PubMed: 9143490. 9143490 10.1006/geno.1997.4642

[15] Stone SL, Hauksdóttir H, Troy A, Herschleb J, Kraft E et al. (2005) Functional analysis of the RING-type ubiquitin ligase family of Arabidopsis. Plant Physiol 137: 13-30. doi:10.1104/pp.104.052423. PubMed: 15644464. 15644464 10.1104/pp.104.052423 PMC548835

[16] Den Herder G, De Keyser A, De Rycke R, Rombauts S, Van de Velde W et al. (2008) Seven in absentia proteins affect plant growth and nodulation in Medicago truncatula. Plant Physiol 148: 369-382. doi:10.1104/pp.108.119453. PubMed: 18599652. 18599652 10.1104/pp.108.119453 PMC2528092

[17] Kim Y-S, Ham B-K, Paek K-H, Park C-M, Chua N-H (2006) An Arabidopsis homologue of human seven-in-absentia-interacting protein is involved in pathogen resistance. Mol Cells 21: 389-394. PubMed: 16819302. 16819302

[18] Callis J, Vierstra RD (2000) Protein degradation in signaling. Curr Opin Plant Biol 3: 381-386. doi:10.1016/S1369-5266(00)00100-X. PubMed: 11019805. 11019805 10.1016/s1369-5266(00)00100-x

[19] Linke B, Nothnagel T, Börner T (2003) Flower development in carrot CMS plants: mitochondria affect the expression of MADS box genes homologous to GLOBOSA and DEFICIENS. Plants J For Cell Molecular Biol 34: 27-37. doi:10.1046/j.1365-313X.2003.01703.x. PubMed: 12662306. 10.1046/j.1365-313x.2003.01703.x 12662306

[20] Landschütze V, Willmitzer L, Müller-Röber B (1995) Inhibition of flower formation by antisense repression of mitochondrial citrate synthase in transgenic potato plants leads to a specific disintegration of the ovary tissues of flowers. EMBO J 14: 660-666. PubMed: 7882969. 7882969 10.1002/j.1460-2075.1995.tb07044.x PMC398129

[21] Geisler DA, Päpke C, Obata T, Nunes-Nesi A, Matthes A et al. (2012) Downregulation of the δ-subunit reduces mitochondrial ATP synthase levels, alters respiration, and restricts growth and gametophyte development in Arabidopsis. Plant Cell 24: 2792-2811. doi:10.1105/tpc.112.099424. PubMed: 22805435. 22805435 10.1105/tpc.112.099424 PMC3426115

[22] Rius SP, Casati P, Iglesias AA, Gomez-Casati DF (2008) Characterization of Arabidopsis Lines Deficient in GAPC-1, a Cytosolic NAD-Dependent Glyceraldehyde-3-Phosphate Dehydrogenase. Plant Physiol 148: 1655-1667. doi:10.1104/pp.108.128769. PubMed: 18820081. 18820081 10.1104/pp.108.128769 PMC2577239

[23] Busi MV, Gomez-Lobato ME, Rius SP, Turowski VR, Casati P et al. (2011) Effect of mitochondrial dysfunction on carbon metabolism and gene expression in flower tissues of Arabidopsis thaliana. Molecular Plants 4: 127-143. doi:10.1093/mp/ssq065. PubMed: 20978083. 10.1093/mp/ssq065 20978083

[24] Czechowski T, Stitt M, Altmann T, Udvardi MK, Scheible WR (2005) Genome-Wide Identification and Testing of Superior Reference Genes for Transcript Normalization in Arabidopsis. Plant Physiology 139: 5-17 10.1104/pp.105.063743 PMC1203353 16166256

[25] Thomas B (2006) Light signals and flowering. J Exp Bot 57: 3387-3393. doi:10.1093/jxb/erl071. PubMed: 16980594. 16980594 10.1093/jxb/erl071

[26] Bergink S, Jaspers NGJ, Vermeulen W (2007) Regulation of UV-induced DNA damage response by ubiquitylation. DNA Repair (Amst) 6: 1231-1242. doi:10.1016/j.dnarep.2007.01.012. PubMed: 17363340. 17363340 10.1016/j.dnarep.2007.01.012

[27] Ulm R, Baumann A, Oravecz A, Máté Z, Adám E et al. (2004) Genome-wide analysis of gene expression reveals function of the bZIP transcription factor HY5 in the UV-B response of Arabidopsis. Proc Natl Acad Sci U S A 101: 1397-1402. doi:10.1073/pnas.0308044100. PubMed: 14739338. 14739338 10.1073/pnas.0308044100 PMC337064

[28] Radivojac P, Vacic V, Haynes C, Cocklin RR, Mohan A et al. (2010) Identification, analysis, and prediction of protein ubiquitination sites. Proteins 78: 365-380. doi:10.1002/prot.22555. PubMed: 19722269. 19722269 10.1002/prot.22555 PMC3006176

[29] Andrade MA, Chacón P, Merelo JJ, Morán F (1993) Evaluation of secondary structure of proteins from UV circular dichroism spectra using an unsupervised learning neural network. Protein Eng 6: 383-390. doi:10.1093/protein/6.4.383. PubMed: 8332596. 8332596 10.1093/protein/6.4.383

[30] Winter D, Vinegar B, Nahal H, Ammar R, Wilson GV et al. (2007) An "Electronic Fluorescent Pictograph" Browser for Exploring and Analyzing Large-Scale Biological Data Sets. PLOS ONE 2: e718. 17684564 10.1371/journal.pone.0000718 PMC1934936

[31] Tohge T, Kusano M, Fukushima A, Saito K, Fernie AR (2011) Transcriptional and metabolic programs following exposure of plants to UV–B irradiation. Plant signaling &amp. Behaviour 6: 1987-1992. 10.4161/psb.6.12.18240 PMC3337192 22112450

[32] Huang X, Ouyang X, Yang P, Lau OS, Li G et al. (2012) Arabidopsis FHY3 and HY5 positively mediate induction of COP1 transcription in response to photomorphogenic UV-B light. Plant Cell 24: 4590-4606. doi:10.1105/tpc.112.103994. PubMed: 23150635. 23150635 10.1105/tpc.112.103994 PMC3531854

[33] Ben-Saadon R, Zaaroor D, Ziv T, Ciechanover A (2006) The polycomb protein Ring1B generates self atypical mixed ubiquitin chains required for its in vitro histone H2A ligase activity. Mol Cell 24: 701-711. doi:10.1016/j.molcel.2006.10.022. PubMed: 17157253. 17157253 10.1016/j.molcel.2006.10.022

[34] Wang C, Deng L, Hong M, Akkaraju GR, Inoue J et al. (2001) TAK1 is a ubiquitin-dependent kinase of MKK and IKK. Nature 412: 346-351. doi:10.1038/35085597. PubMed: 11460167. 11460167 10.1038/35085597

[35] Yin Q, Lin S-C, Lamothe B, Lu M, Lo Y-C et al. (2009) E2 interaction and dimerization in the crystal structure of TRAF6. Nat Struct Mol Biol 16: 658-666. doi:10.1038/nsmb.1605. PubMed: 19465916. 19465916 10.1038/nsmb.1605 PMC2834951

[36] Baud V, Liu ZG, Bennett B, Suzuki N, Xia Y et al. (1999) Signaling by proinflammatory cytokines: oligomerization of TRAF2 and TRAF6 is sufficient for JNK and IKK activation and target gene induction via an amino-terminal effector domain. Genes Dev 13: 1297-1308. doi:10.1101/gad.13.10.1297. PubMed: 10346818. 10346818 10.1101/gad.13.10.1297 PMC316725

[37] Laemmli UK (1970) Cleavage of structural proteins during the assembly of the head of bacteriophage T4. Nature 227: 680-685. doi:10.1038/227680a0. PubMed: 5432063. 5432063 10.1038/227680a0

[38] Bollag DM, Rozycki MD, Edelstein SJ (1996). roteins Methods: 415.

[39] Bradford MM (1976) A rapid and sensitive method for the quantitation of microgram quantities of protein utilizing the principle of protein-dye binding. Anal Biochem 72: 248-254. doi:10.1016/0003-2697(76)90527-3. PubMed: 942051. 942051 10.1016/0003-2697(76)90527-3

[40] Wertz IE, O’Rourke KM, Zhang Z, Dornan D, Arnott D et al. (2004) Human De-etiolated-1 regulates c-Jun by assembling a CUL4A ubiquitin ligase. Science (New York, NY) 303: 1371-1374. doi:10.1126/science.1093549. PubMed: 14739464. 10.1126/science.1093549 14739464

[41] Lario LD, Ramirez-Parra E, Gutierrez C, Casati P, Spampinato CP (2011) Regulation of plant MSH2 and MSH6 genes in the UV-B-induced DNA damage response. J Exp Bot 62: 2925-2937. doi:10.1093/jxb/err001. PubMed: 21307385. 21307385 10.1093/jxb/err001

